# Advances in Precision Medicine: Tailoring Individualized Therapies

**DOI:** 10.3390/cancers9110146

**Published:** 2017-10-25

**Authors:** Kyle B. Matchett, Niamh Lynam-Lennon, R. William Watson, James A. L. Brown

**Affiliations:** 1 Centre for Cancer Research and Cell Biology, Queen’s University Belfast, 97 Lisburn Road, Belfast BT9 7AE, UK; k.matchett@qub.ac.uk; 2 Department of Surgery, Trinity Translational Medicine Institute, Trinity College Dublin, College Green, Dublin 2, D08 W9RT, Ireland; LYNAMLEN@tcd.ie; 3 School of Medicine, Conway Institute of Biomolecular and Biomedical Research, University College Dublin, Dublin 4, D04 V1W8, Ireland; william.watson@ucd.ie; 4 Discipline of Surgery, School of Medicine, The Lambe Institute for Translational Research, National University of Ireland Galway, Galway H91 YR71, Ireland

**Keywords:** Ireland Cancer Meeting, personalized, PDX, avatar, precision medicine, profiling, Xenografts

## Abstract

The traditional bench-to-bedside pipeline involves using model systems and patient samples to provide insights into pathways deregulated in cancer. This discovery reveals new biomarkers and therapeutic targets, ultimately stratifying patients and informing cohort-based treatment options. Precision medicine (molecular profiling of individual tumors combined with established clinical-pathological parameters) reveals, in real-time, individual patient’s diagnostic and prognostic risk profile, informing tailored and tumor-specific treatment plans. Here we discuss advances in precision medicine presented at the Irish Association for Cancer Research Annual Meeting, highlighting examples where personalized medicine approaches have led to precision discovery in individual tumors, informing customized treatment programs.

## 1. Introduction

Precision oncology combines state-of-the-art molecular profiling (genetic, cellular and molecular information) of each patient with established clinical-pathological indexes to inform individual patient’s diagnostic and prognostic risk profile. This improves clinical stratification and places each patient at the center of an individualized, therapeutic treatment plan [1]. Precision oncology offers a range of approaches that is revolutionizing the management and treatment of cancer [2,3,4].

Here we discuss key techniques employed at the forefront of personalized medicine, presented at the 2017 Irish Association for Cancer Research (IACR) Annual Meeting. A range of key precision medicine approaches were used and often combined, providing key insights into the complex molecular signaling observed in individual tumors and revealing novel treatment options for individual patients. Crucially, these approaches can predict therapeutic responses in patients, in near real-time, providing key pre-clinical insights that can be used to guide clinical therapeutic decisions.

Successful oncology drug development has been sub-optimal, manifested by the very low percentage of new agents that ultimately achieve regulatory approval. This high failure is partially due to the inability of standard models to accurately predict clinical response, or tailor chemotherapy to specific high benefit patient groups. Patient avatars (personalized tumorgraft models or Patient Derived Xenografts: PDXs) offer a potential solution to some of these challenges maintaining a tumors heterogeneity (a significant factor for personalized medicine [5]), molecular architecture and therapeutic responses ex vivo [6,7], allowing treatment options to be tested, informing clinical therapeutic choices.

## 2. Ex Vivo Models Predict Treatment Responses

Professor David Sidransky (Johns Hopkins University, Baltimore, Washington, DC, USA) combined mutation profiling with a propagating PDX model, identifying individual tumors driver mutations. Whole exome next-generation sequencing (WES) of 4 PDX models and corresponding parental tumors validated their model, demonstrating nearly identical mutational landscapes (unpublished data [8]). WES of 237 early passage PDX tumors (including breast, ovarian, head and neck, colorectal and lung cancers) identified key driver mutations and displayed mutational fidelity to samples in the TCGA database [9] (and unpublished data [8]). This driver mutation dataset represents one of the largest genomic analyses of PDX models to date and allows tumor-specific targeted treatments to be selected and tested ex vivo.

Previously, small retrospective patient avatar studies demonstrated that chemotherapeutic responses mimicked patients’ clinical outcomes [10,11]. Professor Sidransky discussed a precise evaluation of real-time correlations between the clinical responses of a patient’s tumor and their matched PDXs. PDXs were established from 92 patients (from five solid tumor types) and the patient and PDX treatment responses evaluated. Analyzing 129 clinical correlations (including patients with multiple treatments) revealed that PDX tumor growth regression accurately paralleled patient clinical responses. A significant association was observed between patient and PDX treatment responses in 87% of the therapeutic outcomes (subsequently published, [12]). Importantly, the PDX models faithfully predicted outcomes before clinical presentation, allowing multiple treatment options (based on mutational profiling) to be explored ex vivo, before clinical implementation. This demonstrates that combining PDX models and mutational profiling allows novel treatments to be selected and tested, paving the way for clinical implementation.

## 3. Real-Time Monitoring of Treatment Responses and Ex Vivo Guided Personalized Treatments

In melanoma, advancements in our understanding of both the genetic landscape and the role of the immune system has enabled the development of more effective targeted therapies and immune checkpoint inhibitors. However, resistance to these treatments is still a significant clinical problem. Professor Richard Marais’ group (Cancer Research UK, Manchester Institute; Manchester, UK) performed WES of plasma-derived circulating tumor DNA (ctDNA), analyzing 364 samples from 214 melanoma patients. Monitoring of BRAF and NRAS driver mutations prospectively predicted responses to targeted and immunotherapies more accurately than serum LDH [13]. A separate WES study analyzed the response of metastatic vaginal mucosal melanoma to sequential targeted, immuno- and chemotherapy. Circulating cell-free DNA (cfDNA) analysis revealed distinct subclonal tumor responses, identifying two tumor subclones, imatinib sensitive (harboring a KIT mutation) and imatinib resistant (KIT wild-type). While each subclone responded differently to immunotherapy, both responded to carboplatin/paclitaxel [14]. Targeted sequencing of ctDNA or cfDNA identified de novo mutations associated with treatment resistance, highlighting WES as a clinical tool for real-time monitoring of treatment responses and identification of resistance mechanisms.

Establishing PDX models within a short timeframe (a mean latency of 49 days and an engraftment rate of 72%), and combining WES profiling of tumors, allowed individualized clinically relevant first- and second-line treatments to be tested and validated in clinically relevant timeframes, positively influencing treatment decisions. In patients with advanced disease, tumors are often inaccessible, which makes the establishment of PDX models problematic. Building on previous work in small-cell lung cancer [15], circulating tumor cell-derived xenografts (CDXs) were established from late stage melanoma patients [13]. The CDXs demonstrated similar genomic profiles and treatment responses to that of the patient’s original tumor, highlighting CDX models as novel tool to predict and monitor individual patient responses in late stage disease.

## 4. Metabolic Profiling Enhances Mutational Profiling

Each tumor’s metabolic profile (low-molecular-weight metabolites and intermediates) influences its dynamic response to treatment. Dr. Ian Mills group (Queen’s University Belfast, Belfast, Northern Ireland, UK) focuses on defining how the interplay between tumor metabolic profiles and mutational and epigenetic alterations regulates gene networks. This offers new approaches to effectively stratify Prostate Cancer (PCa) by identifying regulatory hubs controlling adaptive responses that provide novel targets for enhancing responses to conventional therapy. The androgen receptor (AR) is required for both normal prostate gland development and at all stages in PCa progression, where the AR acts as a transcription factor sustaining the expression of genes facilitating reprogramming towards a cancer phenotype [16]. The prostate gland is a regulator of metabolic process, a net secretor of citrate derived from the TCA cycle and also polyamines such as spermine, supported by one-carbon metabolism [17]. Significant spectroscopic data from clinical samples (biological fluids and tissue samples) has reported reductions in these metabolites as prostate cancer develops with concomitant increases in lactate (a by-product of glycolysis) and phosphocholine in tissue samples. Extending this concept further, they investigated genetic alterations (mutational and epigenetic) in metabolic genes in clinical datasets (including TCGA). Previous studies found that mutations in TCA cycle enzymes, such as isocitrate dehydrogenase (IDH) (1–2% of PCa), could restrict alpha-ketoglutarate production and consequently TET (Ten-eleven translocation) activity, contributing to increased DNA methylation (5-methylcytosine) [18]. Alpha-ketoglutarate derived from the TCA cycle is used a cofactor for a class enzyme, TETs methylcytosine dioxygenases that converts epigenetically silenced DNA (5-methylcytosine modified) to 5-hydroxymethylcytosine and can in some contexts act as transcription factor recruitment sites [19]. A meta-analysis of TCGA data found a strong correlation between IDH mutation and DNA hypermethylation (unpublished data [20]). The importance of DNA hypermethylation as a ‘stem’ biomarker in PCa was supported by the number of high-incidence hypermethylation events impacting gene promoters (*HES5*, *TAACC2*, *RARB*, *GSTP1*) [21], in stark contrast to the significant mutational heterogeneity/low-incidence somatic point mutations affecting individual genes in clinical samples [22]. Cataloguing these mutations and their metabolic effects provides a key resource for referencing individual tumor profiles against, informing and guiding personalized clinical treatment decisions.

Dr. Luca Magnani’s group (Imperial College London, London, UK) demonstrated the importance of combining metabolic and mutation profiling in recurrent breast cancer patients. Endocrine therapies represent the gold standard for the treatment of breast cancer as first line treatments following curative surgery. Importantly, these therapies target, directly or indirectly, estrogen receptor alpha (ERα). Aromatase inhibitors (AIs) block estrogen production in various sites of the body, while Tamoxifen directly antagonizes estrogen receptor (ER) activation. Their similar method of action suggested that any resistance mechanisms would likewise be similar. However, recent evidence in metastatic patients revealed AI resistant tumors were particularly enriched for ER mutations, which were demonstrated to confer resistance [23,24]. Moreover, drug-specific resistance mechanisms were not limited to ER mutations, as cholesterol biosynthesis can directly fuel ER activation via alternative ligands. Examining a cohort of matched primary-metastatic relapses treated uniquely with either Tamoxifen or AIs they observed AI-treated patients had significant amplification of the aromatase gene *CYP19A1* and this amplification event was rare in patients treated with Tamoxifen [25]. This highlighted the importance of profiling tumor recurrence, allowing the monitoring of acquired resistance mechanisms and tailoring of treatments.

Combining recent advances allowing metabolic profiling and mutational analysis of tumors has revealed dynamic responses to treatment, informing both personalized treatment options and disease progression.

## 5. Real Time Mutational Profiling Led Risk Stratification

Professor Irene Roberts’ (University of Oxford, Oxford, UK) research focuses on real time mutational profiling of primary leukemia cells (myeloid and lymphoid) at each stage of leukemia evolution in downs syndrome (DS) children to stratify patient risk [26]. These leukemias each have distinct molecular and biological features as well as a progressive pattern of early onset [27]. Myeloid Leukemia of DS (ML-DS) originates in fetal HSPC and presents as a neonatal preleukaemic syndrome, characterized by abnormal megakaryopoiesis, known as Transient Abnormal Myelopoiesis (TAM) followed in some cases by full ML-DS before 4 years old. In both ML-DS and TAM, the leukemic cells have acquired N-terminal truncating mutations in the transcription factor gene *GATA1* which result in exclusive production of a short GATA1 protein (Gata1s) with altered functional properties [26]. Using an on-going prospective study of the hematological and *GATA1* mutation status in >400 children with DS (from birth to age 4 years; Oxford Imperial Down Syndrome Cohort: OIDSC) allowed the identification of the DS group at high risk of transformation to ML-DS. Conversely, no children without a *GATA1* mutation (at birth) developed ML-DS (unpublished data [28]), marking the low risk group. Importantly the mutational profiling performed in conjunction with the risk stratification has identified potential genes (including the Hsa21 genes *ERG*, *DYRK1A*, *HMGN1*, *USP16* and *miR125b*) and pathways that represent potential future therapeutic targets for the treatment of childhood leukemia.

## 6. Circulating Biomarker Led Detection of High-Risk Patients

Circulating biomarkers can allow the calculation of patient risk, tumor mass and heterogeneity and the prediction/monitoring of treatment outcomes, in real-time. The advent of fast, sensitive and reliable assays (such as reverse transcriptase quantitative PCR: RTqPCR) allows the detection of tumor-derived biomarkers in blood and related bio-fluids, and lays the foundation for the introduction of rapid biomarker based assays into clinical practice. Neuroblastoma often presents with tumor cells in the bone marrow and accurate disease detection is essential for prognostication and assessment of disease response [29]. Professor Sue Burchill’s group (University of Leeds, Leeds, UK) pioneered key improvements in the sensitivity and specificity of disease detection using RTqPCR, detecting a single tumor cell in over 10 million normal cells [30]. In a prospective study of 290 children with stage 4 neuroblastoma, RTqPCR detection of neuroblastoma-specific mRNAs in blood enabled the early identification of drug refractory disease and monitoring of treatment response and progression in real time [31]. High levels of specific mRNA (*TH*, *PHOX2B*, or *DCX*) in peripheral blood or bone marrow at diagnosis strongly predicted for worse event-free survival (EFS) and overall survival (OS). Importantly, following induction therapy high levels of these specific mRNAs predicted worse EFS and OS in bone marrow, but not in peripheral blood [31]. The predictive power of these mRNAs at diagnosis reveals how their quantification is a biomarker for early identification of stage 4 disease, in children for whom current treatment will fail and who may be candidates for alternative novel experimental therapeutics. Introducing this test into current clinical practice would provide logistical, financial, and clinical advantages, improving patient outcomes.

The introduction of trastuzumab-containing chemotherapy over a decade ago dramatically altered the natural history of HER2-positive early stage (non-metastatic) breast cancer. However, despite excellent results, some patients fail to respond to trastuzumab, or relapse after initial curative therapy. ICORG 10-05 (TCHL) is a randomized phase II, three-arm, clinical trial conducted by the Irish Clinical Oncology Research Group (formerly ICORG, now Cancer Trials Ireland, Dublin, Ireland) and presented by Dr. Giuseppe Gullo (St Vincent’s Hospital, Dublin, Ireland). This trial compared a neoadjuvant strategy of docetaxel and carboplatin chemotherapy with either a dual HER2-blockade (lapatinib and trastuzumab) or single HER2-blockade (lapatinib or trastuzumab alone [32]). The study profiled tumor samples from responders and non-responders (collected before, during and after treatment). A number of potential serum biomarkers of response to trastuzumab and/or lapatinib were measured, including the HER2 extracellular domain (ECD). Peripheral blood mononuclear cells (PBMCs) implicated in antibody-dependent cell-mediated cytotoxicity (ADCC) response to trastuzumab were quantified by flow cytometry in the patient samples. Additionally, plasma was profiled for immune-related biomarkers of response using a Luminex multiplex assay capable of examining up to 40 immune-related factors. The pathological complete response rates after neoadjuvant chemotherapy were 54% and 40% in the TCH and TCHL, respectively, with associated survival data pending (unpublished data [33]). ICORG 10-5 represents an excellent example of clinical trials combining with molecular discovery in a precision medicine approach, to understand tumor responses and inform treatment choices.

Tailoring personalized treatments to recurrent, resistant tumors necessitates understanding drug resistance in heterogeneous tumors. Metastatic cancers challenged with targeted agents promote growth of resistant cell populations and consequently targeted therapies are often only transiently effective. It is essential to design future clinical trials to validate strategies to either prevent or overcome this resistance by investigating how to overcome disease recurrence following treatment with targeted agents [34]. Addressing this requires a deeper understanding of how tumors evolve following treatment [35]. Professor Alberto Bardelli’s group (University of Torino, Torino, Italy) used colorectal cancer (CRC) as a model system to explore how tumor evolution can control drug resistance. They found clonal dynamics can be monitored in real-time in patient blood, allowing detection of resistant clones before relapses clinically manifest (unpublished data [36]). Furthermore, they discovered that a multistep clonal evolution process driven by progressive increases in drug resistance underlies the development of resistance in cells and patient avatars (PDXs) (unpublished data [36]). Understanding and predicting tumor evolution in response to therapy will allow long-term targeted therapeutic strategies that account for the continuous evolution of cancer cells and using real time monitoring to implement treatment changes before they lose effectiveness. Knowing in advance how CRC cells overcome resistance to EGFR blockade can inform novel precision treatment strategies which anticipate and monitor this process.

## 7. Discussion and Concluding Remarks

A recent genomic study of PDX models of cancer has highlighted some of the challenges in the use and interpretation of results from PDX models, suggesting that PDX models alter the genomic evolution of the implanted cells, which affects their response to therapies [37,38]. Other challenges limiting the widespread use of PDX models include the time taken to generate each individual PDX, the complication of using immune-compromised models (for analysis, interpretation and translation of results), the significant resources required to create and maintain these models, a lack of benchmarking criteria (to monitor and assesses PDX based tumors changes compared to the primary tumor) and a need for standardized clinically-relevant “response” criteria [7].

While patient avatars do not perfectly mimic all aspects of a human tumor response, they do provide a better model for preclinical evaluation of treatment effects (over cell line-based models) and have been demonstrated to provide significant value in guiding real-time clinical treatment decision-making. Patient avatars can reveal critical clinical points (or tumor evolutions), informing treatment choice and timing (particularly relevant for recurrent hard to treat disease).

The use of rapid, high throughput genomic sequencing can provide insights into each tumors’ unique mutational landscape (in real time), stratifying patients and revealing potential therapeutic targets and combined with key techniques (mutational profiling, data analysis, PDXs, metabolic profiling, circulating biomarkers) provides a powerful toolbox for the optimization of personalized therapies. Furthermore, these techniques reveal fundamental mechanisms of cancer progression or treatment resistance, discoveries exploited in turn to improve patient treatment options and ultimately survival.

The annual IACR Meeting [39] in 2017 highlighted the progress in individualized precision medicine gained from applying advances in individualized real-time tumor profiling. Importantly, combining these approaches was demonstrated to be a powerful method for the optimization and monitoring of treatments that can have real-time impacts on patient outcomes, and is the future of medicine.

## Appendix A

**Table A1:** Conference Speaker List.

Speaker	Affiliation
Professor David Sidransky	The Johns Hopkins University School of Medicine, Baltimore, MD, USA
Professor Sue Burchill	Leeds Institute of Cancer and Pathology, University of Leeds, United Kingdom
Professor Irene Roberts	Weatherall Institute of Molecular Medicine, University of Oxford, United Kingdom
Dr. Ian Mills	Centre for Cancer Research and Cell Biology, Queens’ University Belfast, United Kingdom
Dr. Luca Magnani	Imperial College London, United Kingdom
Professor Alberto Bardelli	Candiolo Cancer Institute-FPO and University of Torino, Italy
Dr. Guiseppe Gullo	St Vincent’s University Hospital, Ireland
Dr. Karen Keeshan	Paul O’Gorman Leukemia Research Centre, University of Glasgow, United Kingdom
Professor Tariq Enver	Cancer Institute, University College London, United Kingdom
Professor Raymond Stallings	Royal College of Surgeons in Ireland, Ireland
Professor Richard Marais	Cancer Research UK Manchester Institute, University of Manchester, United Kingdom
Dr. Janusz Krawczyk	Galway University Hospital and National University of Ireland Galway, Ireland
Dr. Alex Eustace	Royal College of Surgeons in Ireland, Ireland
Dr. Declan Soden	Cork Cancer Research Centre, University College Cork, Ireland
Dr. Derek Power	Cork University Hospital and Cork Cancer Research Centre, University College Cork, Ireland
Dr. Annette Byrne	Royal College of Surgeons in Ireland, Ireland
Professor William Gallagher	UCD Conway Institute, University College Dublin, Ireland
